# Pore structure control factors of polyamine-bridged polysilsesquioxanes by sol–gel method and their structure-adsorption properties for Au(III)

**DOI:** 10.1080/14686996.2018.1484657

**Published:** 2018-08-14

**Authors:** Meng Jin, Rao Fu, Changmei Sun, Rongjun Qu, Chunnuan Ji, Ying Zhang, Ying Wang

**Affiliations:** School of Chemistry and Materials Science, Ludong University, Yantai, China

**Keywords:** Polyamine-bridged polysilsesquioxanes, pore structure, sol–gel, synthesis, adsorption, 20 Organic and soft materials (colloids, liquid crystals, gel, polymers), 102 Porous / Nanoporous / Nanostructured materials, 301 Chemical syntheses / processing

## Abstract

A series of bridged polysilsesquioxane (BPS) materials was synthesized by the sol–gel method from 3-chloropropyl trimethoxysilane, diethylenetriamine (DETA) or ethylenediamine. Tetraethyl orthosilicate (TEOS) and/or one of the two templates, hexadecyl trimethyl ammonium bromide (CTAB) or P123, were used in the co-condensation process to construct some of the porous adsorbents. The adsorption of Au(III) was the highest for samples without TEOS, especially for the DETA series with CTAB template. This study elucidates the synthesis and applications of BPS materials.

## Graphical Abstract

**Figure:**
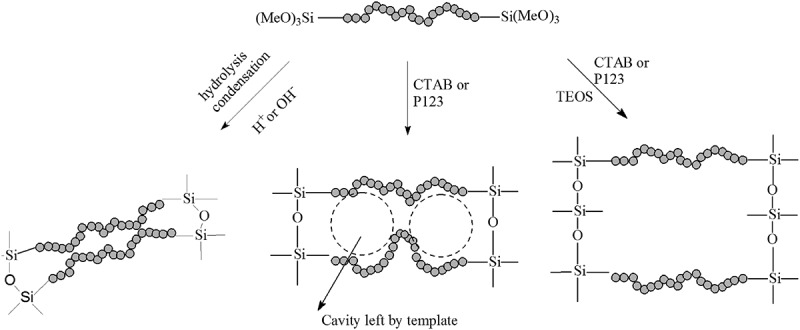

## 1. Introduction

Much attention in the past decade has been given to nanomaterials, as well as their applications in many fields including medicine, physiology, textile, electronics, aerospace and so on [1,2]. Mesoporous silica nanoparticles with particle sizes less than 100 nm and desired properties have been synthesized using various methods for several promising applications in the fields of biomedicine, catalysis and adsorption [3,4]. Among them, adsorption is an economical and efficient method to remove heavy metal ions and it has generated much interest among the people of environmental engineering and science. Researchers think that the techniques of synthesis of advanced adsorbents should be simple and repeatable and the methods underlying the synthesis of such adsorbents should give an opportunity for affecting physicochemical and structure-adsorption properties of the final product. From this point of view, the bridged polysilsesquioxanes (BPSs) are attractive compounds.

BPSs are a class of organic–inorganic hybrid materials prepared by the hydrolysis and polycondensation of organically substituted alkoxysilanes. Generally, BPSs are prepared from monomers that can be represented as (RO)_3_Si-*b*-Si(OR)_3_, where R is the alkyl group and *b* is the organic bridge or spacer. The use of BPSs enables facile preparation of systems of controlled porosity [5,6], because the distance between silicon atoms in the silica network can be readily fine-tuned by varying the rigidity and length of the organic bridge. The textural properties of the final product can be managed by altering the synthetic conditions [7]. Because of these advantages, BPSs are considered an ideal material as adsorbents [8,9]. Porous BPS materials have been prepared from monomers bearing rigid and short organic bridging groups, including ethane, ethylene, as well as small organic aromatics such as thiophene, xylene and benzene [10]. However, there is little research on BPS materials with long and soft bridges containing –NH_2_ or –OH groups, even though such functional groups are expected to contribute to adsorption.

Recently, we synthesized a series of diethylenetriamine-bridged polysilsesquioxanes (DETA-BPSs) by the sol–gel polymerization of the corresponding monomer containing DETA as the soft and long organic bridge [11,12]. It was found that porous BPSs could hardly be obtained through regular sol–gel method due to serious entanglement between organic bridges, unless fully hydrolyzable silanes such as tetramethyl orthosilicate and tetraethyl orthosilicate (TEOS) were added. In this work, we report the synthesis of four series of polyamine-BPSs and examine the factors that control the pore structure of the BPS materials. The adsorption capabilities of these materials for Au(III) were also investigated.

## 2. Experimental details

### 2.1. Chemicals

All reagents were of analytical grade. The silylant agents, 3-chloropropyl trimethoxysilane (CPTS) and TEOS, were purchased from Qufu Wanda Chemicals Factory (Qufu, China). Ethylenediamine (EDA) and DETA were purchased from Laiyang Shuangshuang Chemicals Factory (Yantai, China) and freshly distilled under reduced pressure before use. The poly(ethylene oxide)–poly(propylene oxide)–poly(ethylene oxide) triblock copolymer (P123) was purchased from Sigma-Aldrich Co. Ltd. (Shanghai, China). Hexadecyl trimethyl ammonium bromide (CTAB) and ammonium fluoride (NH_4_F) were purchased from Tianjin Regent Chemicals Co. Ltd. (Tianjin, China). Millipore water was used in all procedures.

### 2.2. Synthesis of monomers

**Scheme 1.**
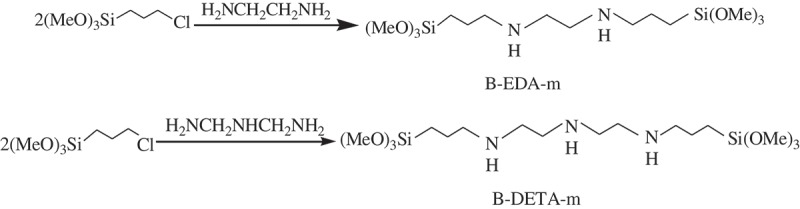
Synthesis of monomers.

The EDA and DETA-bridged monomers, denoted as B-EDA-m and B-DETA-m, respectively, were synthesized as shown in Scheme 1.

Specifically, the solution of EDA (46 mmol, 3 mL) or DETA (46 mmol, 5 mL) in ethanol (100 mL) was prepared in a three-neck flask, and CPTS (92 mmol, 22 mL) was added dropwise. The mixture was maintained under a N_2_ atmosphere with mechanical stirring for 12 h at an elevated temperature of 85 °C. The monomer concentration in the resulting ethanol solution of the bridged monomer was determined gravimetrically as 90.2% and 88.6%.

### 2.3. Synthesis of BPSs without the template or TEOS

In a typical procedure, 4 mL of NH_4_F solution (0.014 g mL^−1^) was added to 40 mL (0.016 mol) B-EDA-m solution or 50 mL (0.016 mol) B-DETA-m solution as prepared in Section 2.2. The self-condensation reaction took place immediately as a transparent gelatinous mixture formed within minutes. The mixture was aged at 70 °C for 4 h and then at 85 °C for 4 days. After filtration, the solid was washed with water, dried in air at room temperature, ground into powder and then solvent-extracted with a mixture of 270 mL of ethanol and 15 mL (37 wt%) HCl for 72 h. The final product was obtained after water rinses and vacuum drying at 60 °C.

### 2.4. Synthesis of BPSs with the template CTAB

CTAB (0.8 g) was completely dissolved into a solution of NaOH (0.12 g in 50 mL warm water) in a three-neck flask. An appropriate amount of the monomer solution (B-EDA-m or B-DETA-m, in ethanol) was added. TEOS was then added as an optional reagent. The molar ratio of TEOS to the monomer was varied to prepare final products with different properties (see Section 3). The mixture was then stirred for 10 min, and an aqueous NH_4_F solution (0.014 g mL^−1^, 4 mL) was added. The reaction mixture was further stirred at room temperature for 24 h to give white precipitates, which were filtered and then maintained in a mixture of ethanol (99.0%, 80 mL) and HCl (37 wt%, 2.0 mL) for 72 h. More details of this procedure can be found in our previous study [13].

### 2.5. Synthesis of BPSs with the template P123

P123 (0.5 g) was completely dissolved into a solution of NaOH (0.12 g in 50 mL warm water) in a three-necked flask. An appropriate amount of the monomer solution (B-EDA-m or B-DETA-m, in ethanol) was added. TEOS was then added as an optional reagent. The mixture was then stirred for 10 min, and an aqueous NH_4_F solution (0.014 g mL^−1^, 4 mL) was added. The reaction mixture was further stirred at room temperature for 24 h to give white precipitates.

The entire reaction mixture including precipitates was transferred to a polypropylene bottle, heated to 70 °C for 4 h and then aged at 85 °C for 4 days. The precipitates were filtered and rinsed with water until no foam formed by P123 could be observed and then dried at room temperature. The collected precipitates were maintained in a mixture of ethanol (99.0%, 270 mL) and HCl (37 wt%, 15 mL) for 72 h and then filtered and dried at 60 °C in a vacuum oven.

### 2.6. Characterization

Infrared (IR) spectra were recorded on a Nicolet MAGNA-IR 550 (Series II) spectrophotometer (Wisconsin, USA). The IR samples were pressed into KBr disks. The spectra were averaged over 32 scans and had a resolution of 4 cm^−1^. The shape and surface morphology of the samples were examined under a Hitachi SU-8010 field emission scanning electron microscope (FESEM) (Tokyo, Japan). The parameters of porous structure were characterized using an automatic physisorption analyzer (ASAP 2020, Micromeritics, Georgia, USA) through the Brunauer–Emmett–Teller (BET) and Barrett–Joyner–Halenda (BJH) methods via N_2_ adsorption at 77 K. Atomic absorption analyses of various metal ions were performed on a flame atomic absorption spectrophotometer (Model 932B, GBC Scientific Equipment Pvt. Ltd., Melbourne, Australia). All samples were subject to elemental analysis on an Elementar VarioEL III instrument (Elementar Analysensysteme GmbH, Frankfurt, Germany) to determine their N contents. X-ray photoelectron spectroscopy (XPS) was recorded on a PHI1600 ESCA photoelectron spectrometer made in Massachusetts, USA. Test conditions were Mg*K*α, 1253.6 eV; power, 200.0 W and resolution, 187.85 eV. Transmission electron microscopy (TEM) images were obtained with a JEM-1230 microscope (Tokyo, Japan) operated at 200 kV. Before TEM measurements, the powder samples were dispersed in ethanol and then dipped and dried on Cu grids.

### 2.7. Adsorption properties

The capacity of the obtained adsorbent materials to adsorb metal ions was measured via a batch adsorption process. Typically, the adsorbent (20 mg) was shaken in the solution of the metal ions (5 mmol L^−1^, 25 mL) for up to 12 h. The concentration of the residual metal ions in the solution was measured by atomic absorption spectroscopy. The capacity of adsorption *W* as calculated according to Equation (1):

(1) $$Q=\frac{\left(C_{0}-C\right)}{W}$$

where *Q* is the adsorbed amount (mmol g^−1^), *V* is the volume of the solution (mL), *W* is the weight of the sample, and *C*_0_ and *C* are the concentration of metal ions at the beginning and at time *t* (mmol mL^−1^), respectively.

## 3. Results and discussion

The adsorbent materials synthesized with the templates were denoted as C-EDA/T-*x* and C-DETA/T-*x* or P-EDA/T-*x* and P-DETA/T-*x*, where C and P refer to CTAB and P123, respectively, T refers to TEOS and *x* refers to the molar ratio of TEOS to B-EDA-m or B-DETA-m in the initial silane mixture. As the control for comparison, the materials synthesized by condensation of B-EDA-m or B-DETA-m without any template or TEOS were denoted as EDA-BPS or DETA-BPS.

### 3.1. Fourier transform infrared spectroscopy

**Figure 1.**
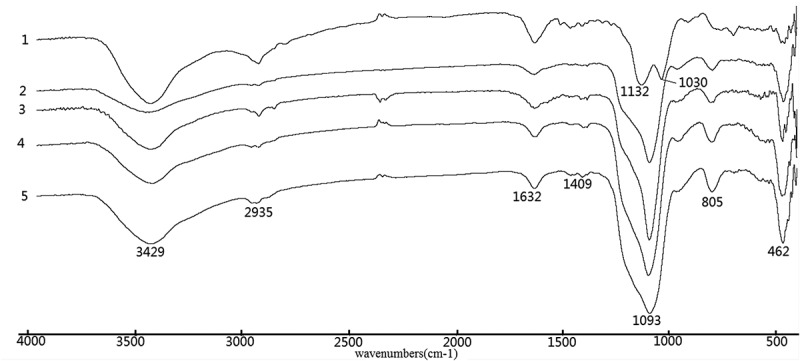
FTIR spectra of EDA-series samples (1) EDA-BPS, (2) C-EDA, (3) C-EDA/T-1, (4) P-EDA and (5) P-EDA/T-1.

**Figure 2.**
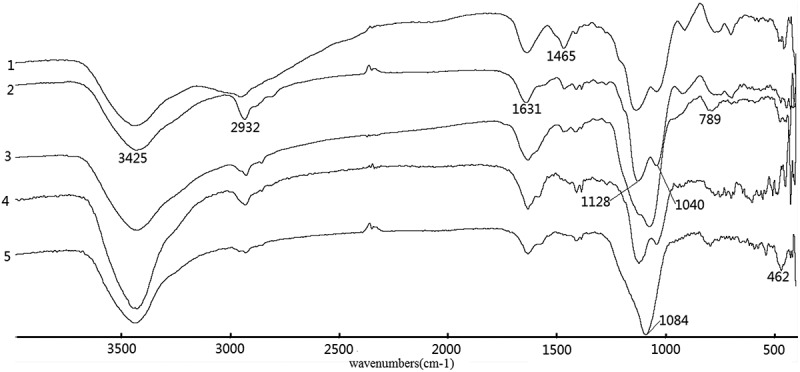
FTIR spectra of DETA-series samples (1) DETA-BPS, (2) C-DETA, (3) C-DETA/T-1, (4) P-DETA and (5) P-DETA/T-1.

Figures 1 and 2 show Fourier transform infrared transmittance spectra of the synthesized materials. Present in the spectra of all materials in Fig. 1 are the characteristic band at ~1093 cm^−1^ with a shoulder at ~1132 cm^−1^ and ~1030 cm^−1^ and the band located at ~805 cm^−1^, which can be attributed to the asymmetric and symmetric vibrations of Si–O–Si framework, respectively [14]. Besides, the bands at ~462 cm^−1^ can be assigned for Si–O–Si bending modes. The –OH bending mode of H_2_O molecules absorbed was found at ~1632 cm^−1^. The weak N–H bending band can be observed at ~1409 cm^−1^ [15]. Another N–H characteristic band typically observed at ~3380 cm^−1^ is not obvious in the spectrum due to the overshadowing effect of the broad O–H band at 3200–3600 cm^−1^.

In Fig. 2, similar characteristic bands can be also found. The band at ~1465 cm^−1^, which can be attributed to Si–O–H, is much stronger in the spectrum of the control (DETA-BPS) than in the other template materials with or without the addition of TEOS and new absorption bands at~2932 and~2935 cm^−1^ are characteristic of the asymmetric and symmetric CH_2_ bands.

It can be deduced that the nitrogen-containing functional groups have been introduced into the silica gel matrix. The IR spectra of other samples are similar to those in Figures 1 and 2, since they all contain similar functional groups.

### 3.2. Factors controlling pore structures

Parameters of pore structures for DETA-series and EDA-series of adsorbent materials are presented in Tables 1 and 2, respectively.

**Table 1.** Parameters of pore structures of DETA-series.

DETA series	BET surface area (m^2^ g^−1^)	BJH desorption cumulative volume of pores (cm^3^ g^−1^)	BJH desorption average pore radius (Å)
DETA-BPS	215	0.68	49.27
C-DETA	215	1.01	78.08
C-DETA/T-1	293	1.22	87.22
C-DETA/T-2	464	0.45	22.77
C-DETA/T-4	392	0.26	37.85
C-DETA/T-6	224	0.19	41.44
C-DETA/T-8	165	0.20	82.65
C-DETA/T-10	136	0.26	51.75
C-DETA/T-20	122	0.17	50.15
P-DETA	300	0.98	56.05
P-DETA/T-1	445	0.49	19.87
P-DETA/T-2	425	0.54	21.64
P-DETA/T-4	277	0.82	50.76
P-DETA/T-6	244	0.90	64.44
P-DETA/T-8	199	0.59	55.85
P-DETA/T-10	205	0.72	67.29
P-DETA/T-20	194	0.42	47.25

**Table 2.** Parameters of pore structures of EDA-series.

EDA series	BET surface area (m^2^ g^−1^)	BJH desorption cumulative volume of pores (cm^3^ g^−1^)	BJH desorption average pore radius (Å)
EDA-BPS	7	0.05	208.14
C-EDA	9	0.03	83.58
C-EDA/T-1	597	0.33	15.13
C-EDA/T-2	419	0.23	18.90
C-EDA/T-4	354	0.38	34.07
C-EDA/T-6	352	0.45	47.88
C-EDA/T-8	149	0.26	65.69
C-EDA/T-10	144	0.32	71.61
C-EDA/T-20	101	0.25	91.64
P-EDA	110	0.55	82.96
P-EDA/T-1	498	0.78	25.08
P-EDA/T-2	415	0.78	29.56
P-EDA/T-4	356	0.57	29.18
P-EDA/T-6	332	0.74	41.91
P-EDA/T-8	292	0.75	35.10
P-EDA/T-10	290	0.74	48.66
P-EDA/T-20	249	0.72	51.96

#### 3.2.1. Organic bridge

For the controls, i.e. materials synthesized without any template or TEOS, the values of BET-specific surface area of EDA-BPS were 7 m^2^ g^−1^, much smaller than that of DETA-BPS (215 m^2^ g^−1^). This may be because the DETA bridge is more flexible and thus favors the formation of larger pores. Another contributing factor may be the larger size of the DETA bridge, which presents more steric hindrance and thus prevents the bridges from tangling up via hydrogen bonds between –OH and –NH groups.

#### 3.2.2. Template

The cationic template CTAB and nonionic template P123 both had a positive effect on the porosity as shown by the increased values of BET-specific surface area of C/P-EDA and C/P-DETA against the controls (EDA-BPS and DETA-BPS) in Tables 1 and 2. The nonionic P123 resulted in significantly greater enhancement in the porosity of the material than the cationic template CTAB.

#### 3.2.3. TEOS

**Scheme 2.**
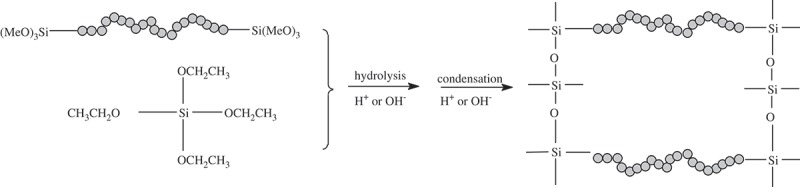
Schematic diagram of interaction of TEOS with bridged monomer.

As a co-monomer, TEOS was added during the synthesis to increase the porosity of the synthesized materials. As shown in Scheme 2, TEOS leads to crosslinks at the Si–O^−^ sites, further widening the distances between the organic bridges. As a result, the hydrogen bonds between the organic bridges are weakened and more porous structures formed.

The molar ratio of TEOS to the monomer was varied between 1:1 and 20:1. Dramatic increases in the values of BET-specific surface area were observed for the EDA series, as shown by a significant increase from 9 m^2^ g^−1^ of C-EDA to 597 m^2^ g^−1^ of C-EDA/T-1, and 110 m^2^ g^−1^ of P-EDA to 498 m^2^ g^−1^ of P-EDA/T-1. This implies that the addition of TEOS effectively reduced the level of entanglement for organic bridges, which made the bridged monomers to be absorbed more freely around the micelle formed by the template molecules resulting in more pores.

However, there was generally a decreasing trend for the values of BET-specific surface area of P-DETA, C-EDA and P-EDA series with the increase of TEOS content. For example, in the C-EDA series, the values of BET-specific surface area decreased gradually from 597 m^2^ g^−1^ of C-EDA/T-1 to 101 m^2^ g^−1^ of C-EDA/T-20. Meanwhile, the pore size increased with the decrease of the values of BET-specific surface area. This may be explained by the increased degree of crosslinking of the O–Si–O matrix with the increase of TEOS content. Micelles formed by the template could be destroyed or pushed out of the O–Si–O network to areas with lower grid density to absorb bridged monomers, resulting in pores with larger size and rather wide pore size distributions (see Figure 7). It is also worth mentioning that the values of BET-specific surface area for series P-EDA and P-DETA were not negatively affected as much as those of series C-EDA and C-DETA. As shown in Table 2, the values of BET-specific surface area decreased from 498 m^2^ g^−1^ of P-EDA/T-1 to 249 m^2^ g^−1^ of P-EDA/T-20 as compared to a decrease from 597 m^2^ g^−1^ of C-EDA/T-1 to 101 m^2^ g^−1^ of C-EDA/T-20. This may be attributed to the different locations of interaction between the templates and the bridged monomers. As illustrated in Schemes 2 and 3, P123 micelles mainly interact with the organic bridge of the monomer and are therefore less affected by the degree of crosslinking of the O–Si–O network. On the other hand, CTAB micelles mainly interact with the Si–O^−^ groups and are more affected by crosslinking.

**Figure 3.**
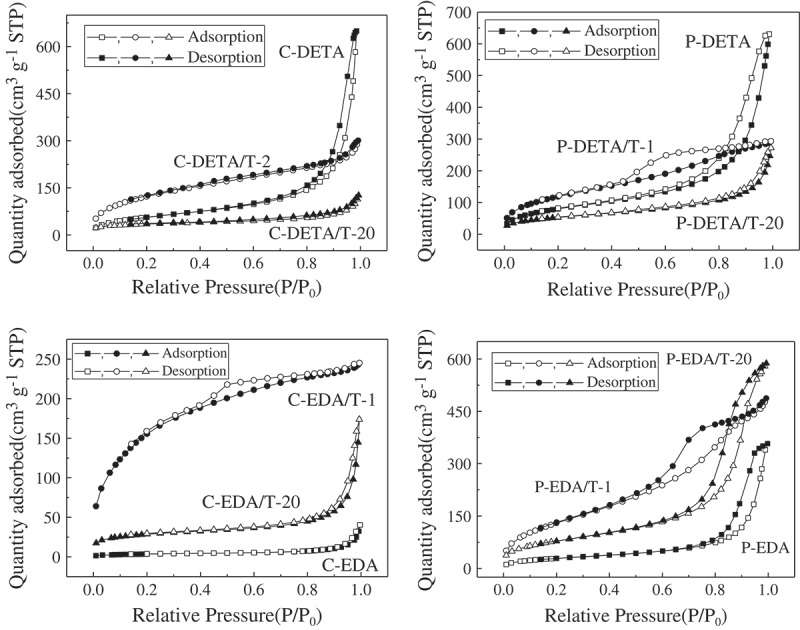
Nitrogen adsorption–desorption isotherms of representative samples of DETA and EDA series.

**Figure 4.**
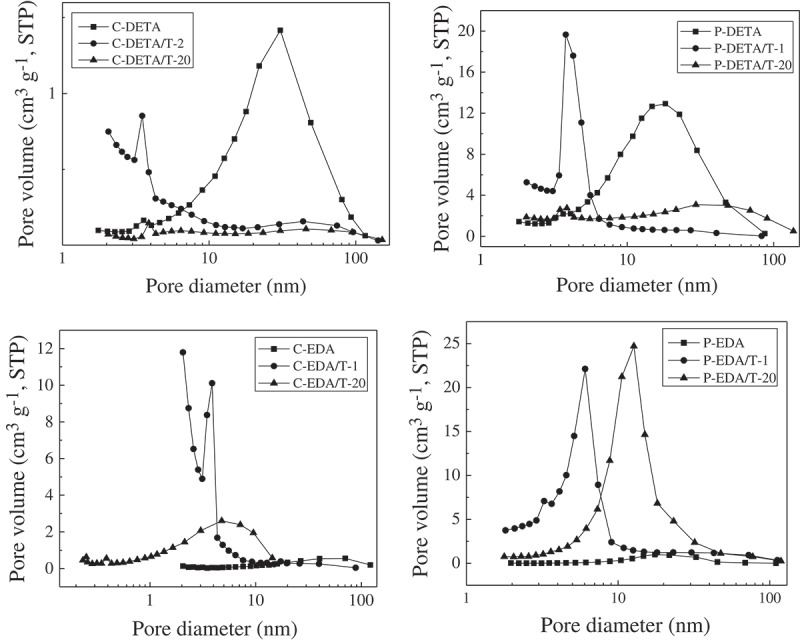
BJH desorption pore size distributions of representative samples of DETA and EDA series.

**Figure 5.**
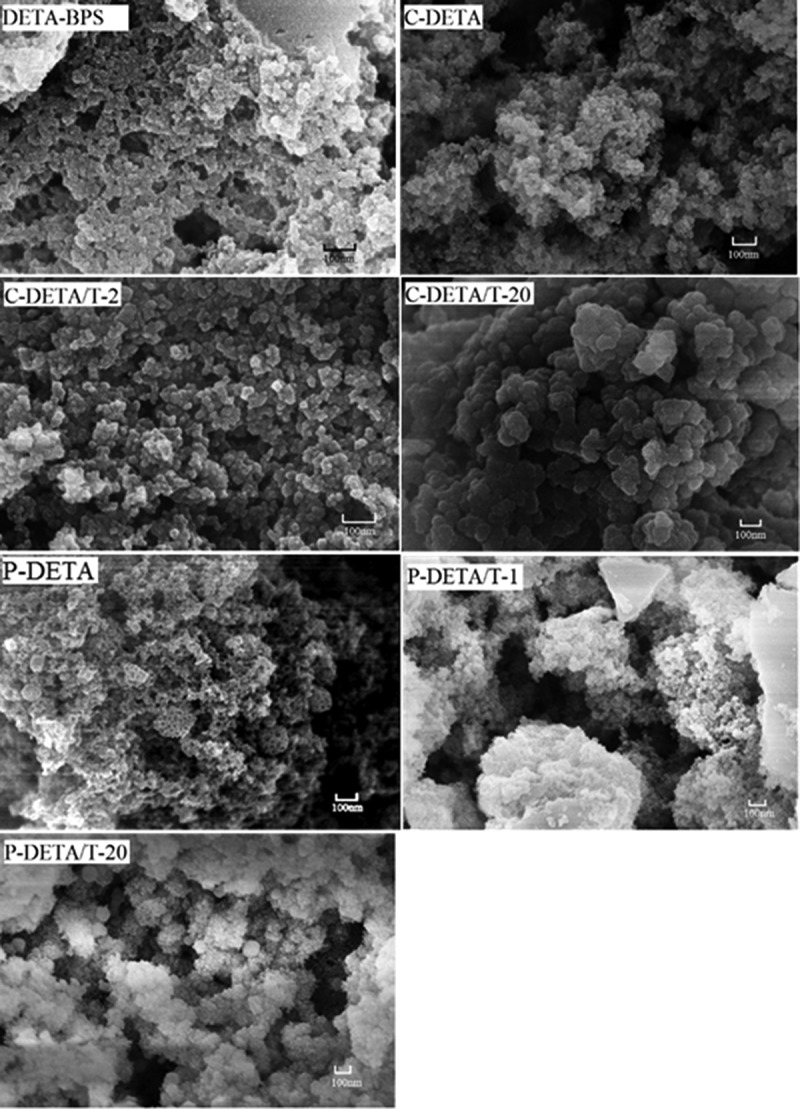
Representative FESEM images of DETA-BPS, C-DETA, C-DETA/T-2, C-DETA/T-20, P-DETA, P-DETA/T-1 and P-DETA/T-20.

**Figure 6.**
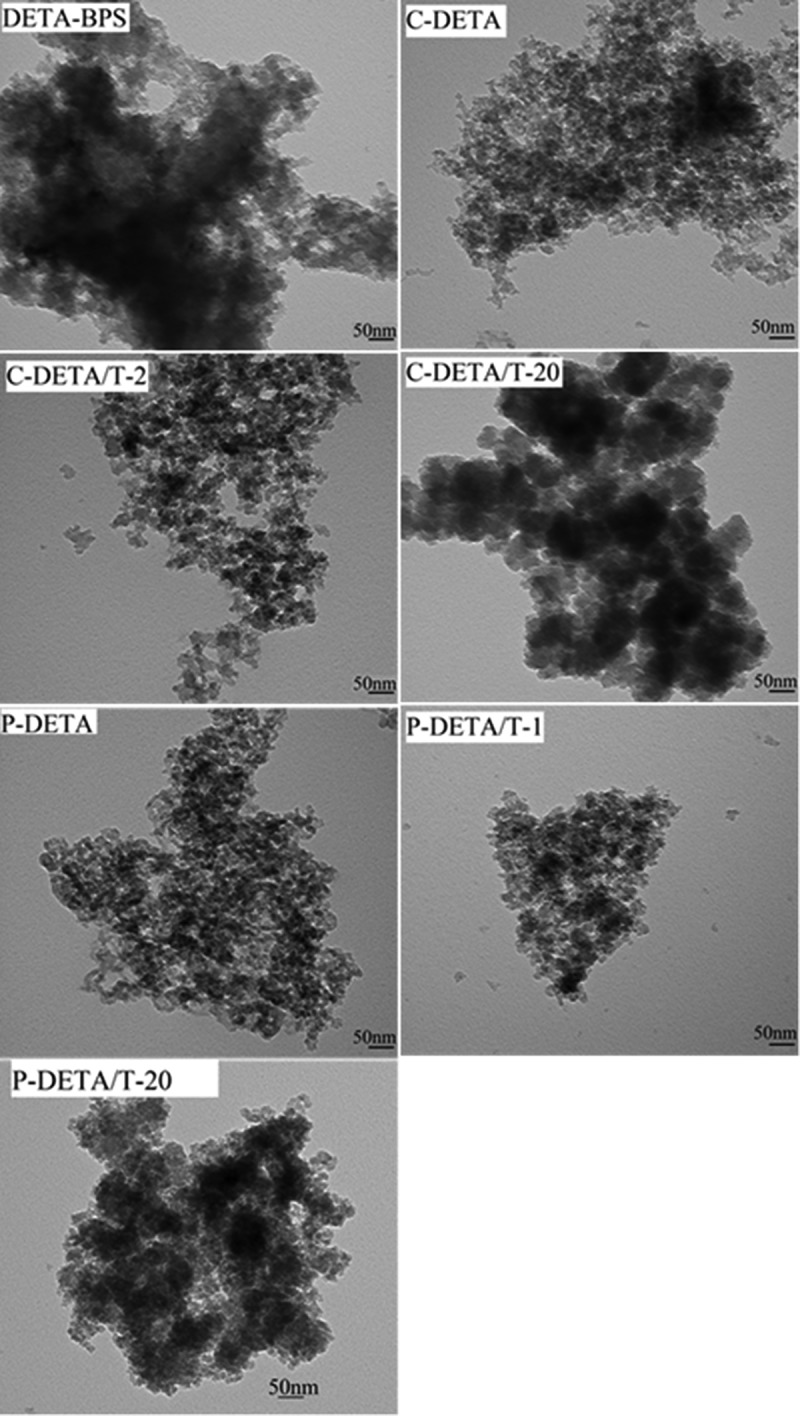
Representative TEM images of DETA-BPS, C-DETA, C-DETA/T-2, C-DETA/T-20, P-DETA, P-DETA/T-1 and P-DETA/T-20.

**Figure 7.**
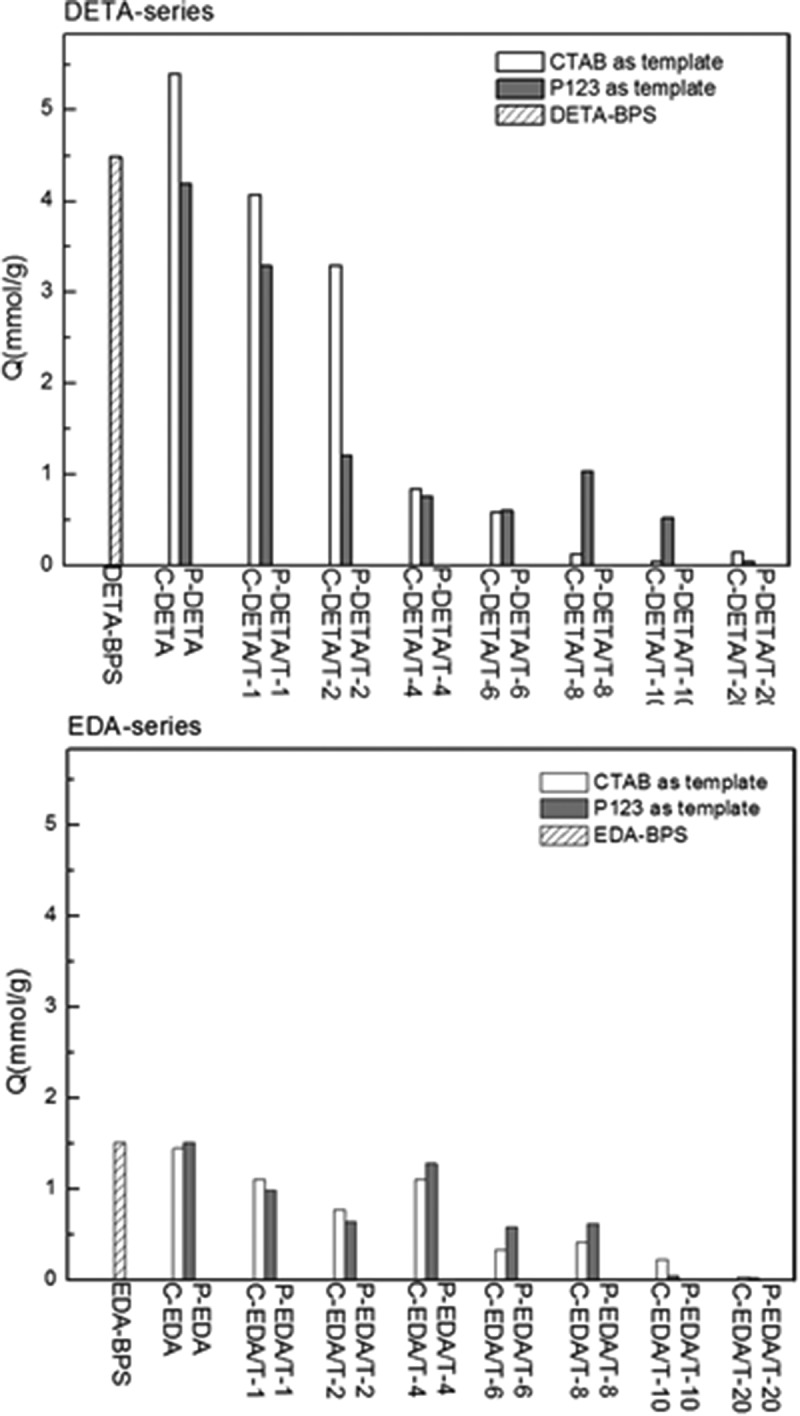
The adsorption capacities of BPS materials of DETA-series and EDA-series for Au(III).

For the C-series, TEOS both negatively affects the values of BET-specific surface area due to increased degree of crosslinking and positively affects the values of BET-specific surface area by reducing the levels of entanglement among the organic bridges. Therefore, there exists an optimal amount of TEOS amount, especially for those bridged monomers with rather long and flexible organic bridges. As shown in Table 1, the optimal molar ratio of TEOS to the monomer is 2:1, at which the highest BET value of 464 m^2^ g^−1^ is achieved in the C-DETA series. A similar scenario was discussed in our previous paper [11] where bridged monomers with even longer organic bridge were used with CTAB as the template.

### 3.3. Nitrogen adsorption–desorption isotherms

Nitrogen adsorption–desorption isotherms of the representative samples are shown in Figure 3. Samples with the highest and lowest the values of BET-specific surface area as well as those without TEOS for each series were selected for analysis. The curves in Figure 3 show an S-shape, which can be classified as type IV according to the IUPAC classification [16]. The curves of samples with the highest the values of BET-specific surface area including C-DETA/T-2, P-DETA/T-2, C-EDA/T-1 and P-EDA/T-1 are different from others. Considering the shapes of hysteresis loops, it can be deduced that more micropores were formed in the samples when TEOS was added. However, with the increasing amount of TEOS, the separation point of isotherm shifted toward higher P/P_0_ values, implying that the number of microporous structures in the material gradually decreases and the number of macroporous structures gradually increases. The presence of pores can be confirmed directly from BJH desorption pore size distribution curves of the samples in Figure 4. The samples with the highest the values of BET-specific surface area have rather narrow desorption pore size distribution in the range of 2–10 nm. With the increasing amount of TEOS, the pore size distribution moved to the larger pore size. By comparing the pore size distribution curves of C-DETA/T-2 and C-EDA/T-1 and P-DETA/T-2 and P-EDA/T-1, it can be concluded that there are more micropores in C-DETA/T-2 and C-EDA/T-1, suggesting that CTAB template is better than P123 in the case where such BPS materials with more micropores are wanted.

The differences between the curves of C-DETA and P-DETA and C-EDA and P-EDA were also studied. For C-DETA and P-DETA, as demonstrated in Section 3.2.1, the DETA bridge is more flexible and favors the formation of larger pores. As shown in Figure 3, both of them have rather high N_2_ adsorption quantities at high P/P_0_ values, indicating the formation of more mesopores and macropores. This can also be confirmed directly from pore size distribution curves in Figure 4, which shows a narrow pore size distribution in the range of 10–100 nm. The porosity of C-EDA and P-EDA is relatively low with a rather wide pore size distribution in the range of 2–100 nm.

### 3.4. FESEM and TEM observation

The FESEM and TEM images of selected DETA-series samples are shown in Figures 5 and 6, respectively. Their surfaces are rough and lumpy and the pores are clearly visible, indicating that the materials are porous. With the addition of the template and TEOS, the materials become loose and porous with a large number of pores. Moreover, the images of C-DETA/T-2 and P-DETA/T-1 with higher values of BET-specific surface area show that they have much denser porous structures than the others.

The TEM images of these samples are shown in Figure 6. The images of DETA-BPS show some black and opaque blocks, while those of porous samples such as C-DETA, C-DETA/T-1, P-DETA and P-DETA/T-1 show loose textures. As the molar amount of TEOS increases to 20:1 with respect to the monomer, the images of C-DETA/T-20 and P-DETA/T-20 start to show black and opaque blocks again. It can be concluded that the mesoporous is not periodical. A similar morphology trend was seen for the EDA-series (not shown).

### 3.5. Au(III) adsorption

Au(III) ion was chosen to evaluate the structure-adsorption properties of adsorbent materials obtained and the results are presented in Figure 7. The elemental analysis results, as listed in Tables 3 and 4, suggest that the content of N decreases with the increase of the amount of TEOS. It is known that both porosity and functionality affect the adsorption capability of an adsorbent. In Figure 7, DETA-series materials exhibit much higher adsorption capacities for Au(III) than EDA-series, especially when the TEOS/monomer molar ratios exceed 4:1. Compared with EDA-BPS, DETA-BPS possesses higher porosity and higher content of N functional groups, consequently resulting in higher adsorption capacity for Au(III). Samples C-DETA and P-DETA possess higher porosity than C-EDA and P-EDA. Although the amounts of N functional groups in them are similar, the former pair shows much higher adsorption capacity for Au(III), suggesting that porosity instead of functionality is the determining factor for adsorption capacity in this case.

**Table 3.** Elemental composition of the DETA-series materials.

DETA series	C%	H%	N%	N (mmol/g)	Au/N
DETA-BPS	26.57	6.62	9.54	6.81	0.66
C-DETA	35.02	6.15	5.93	4.24	1.27
C-DETA/T-1	20.64	4.74	3.12	2.51	1.62
C-DETA/T-2	17.64	3.74	2.82	2.01	1.64
C-DETA/T-4	9.75	2.51	1.33	0.95	0.88
C-DETA/T-6	8.89	2.21	1.01	0.72	0.81
C-DETA/T-8	2.01	0.58	0.92	0.65	0.20
C-DETA/T-10	15.38	3.84	0.85	0.59	0.03
C-DETA/T-20	5.62	1.97	0.56	0.40	0.38
P-DETA	32.91	5.82	5.25	3.75	1.12
P-DETA/T-1	14.70	3.14	2.02	1.44	1.29
P-DETA/T-2	10.99	2.43	1.50	1.07	1.13
P-DETA/T-4	9.75	2.39	2.03	1.45	0.53
P-DETA/T-6	7.86	2.00	1.59	1.14	0.53
P-DETA/T-8	7.22	1.94	1.46	1.04	1.01
P-DETA/T-10	6.66	1.91	1.26	0.90	0.59
P-DETA/T-20	0.14	1.17	0.79	0.56	0.02

**Table 4.** Elemental composition of the EDA-series materials.

EDA series	C%	H%	N%	N (mmol/g)	Au/N
EDA-BPS	25.87	5.68	6.55	4.68	0.32
C-EDA	30.34	0.80	5.26	3.76	0.39
C-EDA/T-1	45.77	8.56	4.25	3.04	0.36
C-EDA/T-2	13.14	2.90	1.14	0.81	0.96
C-EDA/T-4	8.94	2.35	0.82	0.59	1.87
C-EDA/T-6	6.65	0.56	0.66	0.49	0.69
C-EDA/T-8	22.87	5.23	0.60	0.42	0.98
C-EDA/T-10	6.84	1.75	0.58	0.41	0.55
C-EDA/T-20	8.40	1.99	0.53	0.38	0.03
P-EDA	27.95	6.44	7.00	5.00	0.30
P-EDA/T-1	24.88	4.32	2.74	1.96	0.50
P-EDA/T-2	13.51	2.92	1.94	1.39	0.46
P-EDA/T-4	0.05	0.91	0.50	0.36	1.57
P-EDA/T-6	15.91	3.25	0.65	0.42	1.39
P-EDA/T-8	5.90	1.49	0.54	0.39	1.59
P-EDA/T-10	6.62	1.71	0.76	0.54	0.02
P-EDA/T-20	6.09	1.85	0.70	0.50	0.02

As the porosity increases as shown by C-DETA/T-1 and P-DETA/T-1 and C-EDA/T-1 and P-EDA/T-1, the addition of TEOS directly leads to the decrease of N content (an indicator of metal chelating functional groups). Although these batches have the highest values of BET-specific surface area among the respective series, their adsorption capacity still decreases. With the gradual increase of TEOS content, the N content decreases correspondingly, leading to reductions in adsorption capacity for Au(III). It can be concluded that the content of chelating functional group is very important for an adsorbent when appropriate porosity has already been achieved.

Tables 3 and 4 also list Au/N ratio in order to evaluate how many binding sites are occupied. Surprisingly, quite a few values are greater than 1. XPS was adopted to explore what happened. Table 5 shows the binding energy (eV) of DETA-BPS, C-DETA and P-DETA before and after Au(III) adsorption, as these three samples exhibited higher adsorption capabilities for Au(III) than the others. XPS-binding energies of Au(III), Au(I) and Au(0) in references are also listed in Table 5. Compared to the Au_4f_ binding energies of original (87.50 and 91.20 eV), some new binding energies appeared after adsorption for, which related to Au(0) and Au(I). This meant that was an electron acceptor and Au(III) ion might be reduced by –NH groups to Au(0) and Au(I) after adsorption. The high loading capacity is possibly due to the release of adsorbed Au(III) from the resin body in Au(0) or Au(I) form and use of vacant sites by Au(III) in the solution phase that makes an adsorption [of Au(III)]–reduction [to Au(0) or Au(I)]–release [of Au(0)]–adsorption cycle [17]. Similar phenomena also have been found in other materials containing –OH or –NH [18,19].

**Table 5.** XPS binding energies (eV) in DETA-BPS, C-DETA and P-DETA samples before and after Au(III) adsorption, and XPS binding energies of Au(III), Au(I) and Au(0) in references.

	N_1s_	O_1s_	Au _4f_
DETA-BPS	398.67, 401.02	531.67	
DETA-BPS-Au	398.89, 401.20	531.76	84.08, 85.99, 87.73
C-DETA	398.86, 401.33	531.83	
C-DETA-Au	399.67, 401.46	532.07	84.70, 87.03, 88.31, 91.02
P-DETA	398.89, 401.37	531.93	
P-DETA-Au	399.59, 401.37	532.02	84.51, 85.63, 87.01, 88.19, 89.35
Au(III) (in [AuCl_4_]^−^) [20]			87.50, 91.20
Au(I) (in AuCl) [20]			84.35, 88.91
Au(0) (in silicate minerals) [21]			84.00, 87.60

It can also be seen from Fig. 10 that C-DETA exhibited the highest adsorption capabilities for Au(III) among the samples and the saturated adsorption capacity can reach as high as 5.4 mmol g^−1^. The adsorption kinetics of C-DETA for Au(III) showed rapid adsorption in the first 20 min and the adsorption capacity can reach to 3.25 mmol g^−1^ at 20 min. Then the adsorption rate decreased gradually until the adsorption reached equilibrium in about 5 h. For other samples, the adsorption equilibrium time decreases with the increase of TEOS content, which might related to the decrease of N content.

Table 6 gives a brief comparison of Au(III) adsorption capacities of C-DETA and some recently reported materials. As expected, adsorption capacity of C-DETA is much higher than other materials [18,22–35], implying its great potential in noble metal enrichment.

**Table 6.** Saturated adsorption capacities for Au(III) of C-DETA prepared in the present work in comparison with those of recently reported adsorbents.

Adsorbents	Saturated adsorption capacities for Au(III) (mmol g^−1^)	References
Polystyrene-supported 3-amino-1,2-propanediol	1.17	[18]
Graphene oxide	0.55	[22]
Silica hybrid quaternary ammonium salts	0.32	[23]
Silica functionalized by an amino phosphonic acid	1.19	[24]
Silica modified with *N,N*-(octane-1,8-diylidene)di(2-hydroxyl-3,5-dimethylaniline)	0.93	[25]
Silica modified with 6-((2-(2-hydroxy-1-naphthoyl)hydrazono)methyl)benzoic acid	1.03	[26]
Silica functionalized with 3-(2-aminoethylamino)propyl	0.50	[27]
Bayberry tannin on mesoporous silica	3.25	[28]
Polyamidoamine-grafted silica gel	2.45	[29]
Ordered mesoporous silica monoliths	0.90	[30]
PDMC-SNP	0.64	[31]
Alkoxycarbonyl thiourea resin	4.65	[32]
Triazine-triethylenetetramine polymers	5.07	[33]
Modified thiol cotton fiber	0.35	[34]
Polystyrene-supported open-chain crown ether	3.20	[35]
C-DETA	5.40	This work

Caption: adsorbents. PDMC stands for poly(methacryloxyethyltrimethyl ammonium chloride) and SNP for silica nanoparticles.

## 4. Conclusions

DETA bridged monomer (B-DETA-m) and EDA bridged monomer (B-EDA-m) were prepared from the reaction of 3-CPTS with DETA and EDA. A series of polyamine BPSs was synthesized by sol–gel polymerization of the prepared monomers. Two types of templates including P123 and CTAB with a co-monomer TEOS were added during polymerization to increase the porosity of the materials. P123 lead to a more significant increase in the values of BET-specific surface area of EDA-BPS and DETA-BPS than CTAB. When the molar ratio of TEOS/B-DETA-m and TEOS/B-EDA-m was 1/1 or 2/1, the maximum BET surface area of the material was achieved. With the increase of TEOS content, the values of BET-specific surface area began to decrease while the pore size increased. It was shown that too much TEOS generated a dense O–Si–O network resulting in the extrusion or destruction of the template micelles. Au(III) ion was chosen to evaluate the structure-adsorption properties of samples. Samples with the highest the values of BET-specific surface area did not exhibit the highest adsorption capacity among their respective series, indicating that the content of chelating functional group is another very important factor for adsorption. C-DETA exhibited the highest adsorption of Au(III) among all samples, which may have certain values for the recovery of precious metals in water or catalyst supports.
